# Beyond the coronal plane in robotic total knee arthroplasty—Part 2: Combined flexion does not affect outcomes

**DOI:** 10.1002/ksa.12660

**Published:** 2025-03-27

**Authors:** Luca Andriollo, Pietro Gregori, Christos Koutserimpas, Elvire Servien, Cécile Batailler, Sébastien Lustig

**Affiliations:** ^1^ Orthopaedics Surgery and Sports Medicine Department, FIFA Medical Center of Excellence, Croix Rousse Hospital, Hospices Civils de Lyon Lyon North University Hospital Lyon France; ^2^ Ortopedia e Traumatologia Fondazione Poliambulanza Istituto Ospedaliero Brescia Italy; ^3^ Fondazione Policlinico Universitario Campus Bio‐Medico Roma Italy; ^4^ LIBM‐EA 7424, Interuniversity Laboratory of Biology of Mobility Claude Bernard Lyon 1 University Lyon France; ^5^ Univ Lyon, Claude Bernard Lyon 1 University, IFSTTAR, LBMC UMR_T9406 Lyon France

**Keywords:** combined flexion, functional alignment, functional knee positioning, sagittal alignment, total knee arthroplasty

## Abstract

**Purpose:**

Personalized alignment in total knee arthroplasty (TKA) is becoming increasingly widespread, driven in part by advancements in robotic‐assisted surgery. However, true personalization must extend beyond the coronal plane to include sagittal and axial planes. This study investigates the impact of combined flexion (CF) of the femoral and tibial components in robotic‐assisted TKA within functional alignment (FA), also analyzing its variation (ΔCF) from native anatomy and its correlation with functional outcomes and complications.

**Methods:**

A retrospective analysis was conducted on 310 patients who underwent primary TKA using an image‐based robotic system between March 2021 and January 2023. CF was calculated based on tibial slope (TS) and distal femoral flexion (DFF). Patients were stratified into groups based on CF (≤7.5° vs. >7.5°) and CF variation (ΔCF < −3, −3 to 3 and >3). Clinical scores, radiographic parameters and complication rates were analyzed.

**Results:**

Patients with CF ≤ 7.5° had lower preoperative maximum flexion values (*p* = 0.005). No significant differences in complication rates or clinical outcomes were observed between groups. ΔCF were associated with post‐operative coronal alignment changes (mechanical hip–knee–ankle angle and medial proximal tibial angle) but did not impact patient‐reported outcomes. Additionally, subgroup analysis revealed that ΔCF were not linked to differences in implant survival, revision rates or mechanical failure.

**Conclusions:**

Although CF influences knee biomechanics, its direct impact on clinical outcomes remains unclear. It is evident that a personalized approach to sagittal alignment can be an integral component of functional knee positioning.

**Level of Evidence:**

Level III.

**Abbreviations**
AFO: anterior femoral offset
AKPS: Kujala Anterior Knee Pain Scale
AP: anterior‐posterior
BMI: body mass index
CF: combined flexion
CR: cruciate‐retaining
CS: cruciate substituting
DFF: distal femoral flexion
FA: functional alignment
FJS‐12: Forgotten Joint Score
FKP: functional knee positioning
HSS: Hospital for Special Surgery Knee Score
KSS: Knee Society Score
LDFA: lateral distal femoral angle
mHKA: mechanical hip–knee–ankle angle
MPTA: medial proximal tibial angle
PCA: posterior condylar axis
PCL: posterior cruciate ligament
PCO: posterior condylar offset
PS: posterior stabilized
ROM: range of motion
TEA: transepicondylar axis
TKA: total knee arthroplasty
TS: tibial slope
ΔCF: variation in combined flexion
ΔDFF: variation in distal femoral flexion
ΔTS: variation in tibial slope

## INTRODUCTION

In the late 2000s, an out‐of‐the‐box approach to alignment in total knee arthroplasty (TKA) emerged, shifting mechanical alignment (MA) from being the gold standard to merely a standard technique, with other alignment methods becoming increasingly widespread [11, 29, 31].

Driven in part by the rise of robotic surgery, there has been a transition from standardized alignment to personalized alignment strategies [6, 13, 24, 32]. However, it is becoming clear that personalized alignment cannot rely only on the coronal plane. To achieve true personalized alignment, it is important to also consider the axial and sagittal planes [1, 15].

Interest in the axial plane is growing, especially in relation to the patellofemoral compartment, also known as the third space [23]. Anterior knee pain remains a common issue after TKA, even in patients who are overall satisfied. Studies suggest that establishing safe zones and avoiding overstuffing may help improve clinical outcomes [17].

Despite advancements in technology, including robotics, which offer greater precision in TKA component placement, the optimal component position and sagittal limb alignment remain unknown [9, 30].

This study complements the concomitant article by Andriollo et al. [2] in the analysis of the sagittal plane. The aim is to analyze the combined flexion (CF) of the tibial and femoral components, their variation relative to the patient's native anatomy, and their correlation with functional outcomes and complications. The hypothesis is that with a personalized alignment, such as functional alignment (FA) or functional knee positioning (FKP), based on three‐dimensional positioning concepts, the sagittal plane, following individualized principles, does not impact clinical outcomes.

## METHODS

**Figure 1:**
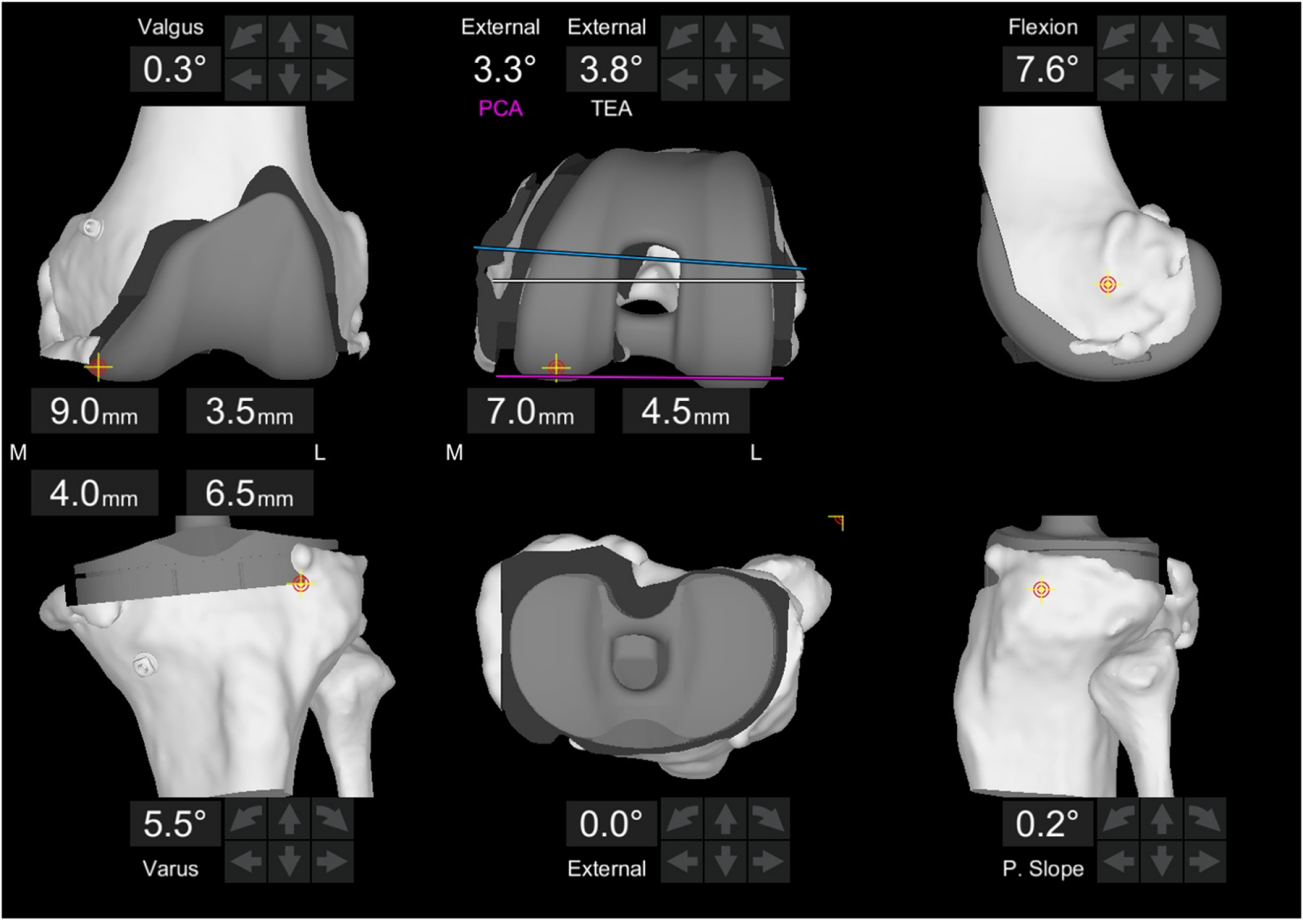
Screenshot from the robotic system of planning for functional knee positioning following the principles of functional alignment, with details of the bone cuts. PCA, posterior condylar axis; TEA, transepicondylar axis.

**Figure 2:**
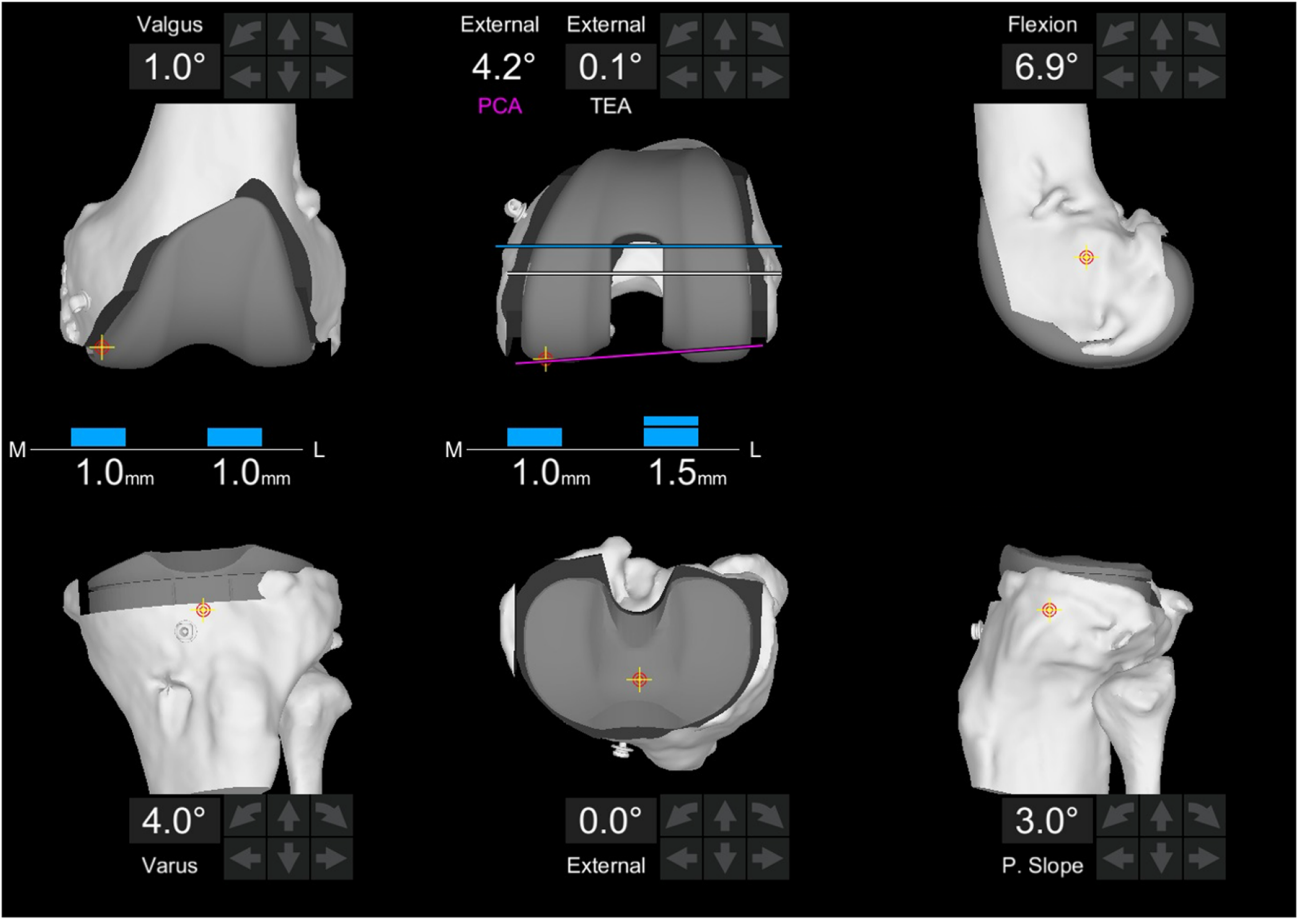
Screenshot from the robotic system of planning for functional knee positioning following the principles of functional alignment, with details of the final laxity based on bone cuts and ligament compliance. PCA, posterior condylar axis; TEA, transepicondylar axis.

This study, designed retrospectively using a prospectively maintained database, included all patients who underwent primary TKA conducted according to FA or FKP principles and performed with the Mako robotic arm‐assisted system (Stryker, Mako Surgical Corp.) between March 2021 and January 2023 (Figures 1 and 2). All patients received a Triathlon Total Knee System implant (Stryker, Mako Surgical Corp.), using either posterior stabilized (PS) or cruciate substituting (CS) inserts.

The procedures were conducted at a single, high‐volume centre specializing in both primary and revision arthroplasty. A consistent surgical technique, aligned with FA principles, was applied to all patients [32, 33].

From the initial pool of 382 patients, those were excluded if preoperative, intraoperative, or follow‐up data required for this study were missing (41 patients), or if a complete robotic data report was unavailable (21 patients).

Demographic data, such as age, gender and body mass index (BMI), were collected. Preoperative evaluations for all patients included measurements of knee range of motion (ROM) and the Knee Society Score (KSS) for both functional and knee‐specific parameters.

Imaging evaluations were performed using anteroposterior, lateral, Rosenberg, sunrise and full‐length weight‐bearing x‐rays. From these, the mechanical hip–knee–ankle angle (mHKA), lateral distal femoral angle (LDFA), medial proximal tibial angle (MPTA), and tibial slope (TS, following the mid‐diaphysis technique) were calculated.

Robotic data on component positioning metrics were obtained from robotic system records. For the femoral component, these include flexion/extension based on the mechanical axis (MA), varus/valgus alignment of the distal cut and rotation relative to the surgical transepicondylar axis (TEA). For the tibial component, varus/valgus alignment of the proximal cut and posterior slope were analyzed.

At the final follow‐up, clinical evaluations included KSS‐knee and KSS‐function, the Forgotten Joint Score (FJS‐12) and the Kujala Anterior Knee Pain Scale (AKPS), as well as data on ROM reported as recurvatum, flexion contracture and maximum flexion.

Data on complications, both septic and aseptic, as well as reoperation and revision rates, were also collected.

Patients were divided into groups to analyze the impact of implant positioning in relation to the sagittal plane. With robotic data on TS and distal femoral flexion (DFF), the CF was calculated. The native TS slope was compared to the implant slope, obtained from robotic data, resulting in an ΔTS value (ΔTS = native TS − implant TS). Regarding the femur, it is known that the native DFF is the angle between the anatomical axis and the MA. The final femoral flexion of the implant, provided by the robot, is the angle between the MA and the implant axis. Consequently, by definition, the final femoral flexion of the implant represents the variation angle of the DFF, with reference to the anatomical axis. The femoral flexion value provided by the robot will hereafter be identified as ΔDFF, indicating the difference in DFF between the native and final states. CF is determined by the flexion of the tibial and femoral components. ΔCF, or the CF difference, was calculated as ΔCF = ΔDFF − ΔTS.

Positive (+) values of CF indicate that the positioning of the tibial and femoral components generates a CF position. A positive (+) value of ΔCF indicates an increase in CF.

Based on CF, the group with values ≤ 7.5° (Group A) was compared to the group with values > 7.5° (Group B). For the evaluation of ΔCF, three groups were compared: <−3 (Group C), between −3 and 3 (Group D) and >3 (Group E).

The groups, within the two respective analyzed parameters, were compared in terms of clinical outcomes, radiographic measurements, robotic data and complication rates.

### Principles of sagittal plane management

The femoral component is placed to closely match the concentricity of Blumensaat's line in cases without trochlear dysplasia while avoiding femoral notching. This method ensures proper component sizing and reduces the risk of patellofemoral overstuffing or understuffing. Femoral flexion ranged from 0° to 10°.

The tibial implant is positioned to replicate the patient's native posterior TS, with a maximum limit of 3° when using a PS implant. Adjustments can be made to balance the flexion gap if needed, while ensuring that the combined femoral‐tibial flexion does not exceed 10°.

The PS insert was preferred in cases of posterior cruciate ligament (PCL) deficiency or significant flexion contracture. With the CS insert, PCL release is performed only in cases of severe flexion limitation with the trial component, but it remains rare.

### Ethical approval

This study followed the ethical guidelines established in the 1964 Declaration of Helsinki and complied with HIPAA standards. The collection and analysis of data were carried out in alignment with the MR004 Reference Methodology set by the French Commission Nationale de l'Informatique et des Libertés (Ref. 2229975V0). Informed consent was obtained from all participants.

### Statistical analysis

A power analysis was conducted using G*Power (*α* = 0.05, *β* = 0.30, medium effect size), identifying a minimum of 44 patients per group was determined to be necessary for comparing variables. All the groups in this study meet the minimum sample. Continuous variables were expressed as mean and standard deviation (SD), while categorical variables were presented as frequency distributions and percentages. The Shapiro–Wilk test was employed to evaluate the normality of the data. For comparisons between two groups, either the *t* test or the Mann–Whitney *U* test was used, depending on whether the data followed a normal distribution. For comparisons among three groups, either ANOVA or the Kruskal–Wallis test was utilized, based on the presence or absence of normality. Post hoc analysis was performed using Bonferroni's test. Categorical variables were analyzed using the chi‐square test.

A 95% confidence interval was applied, and statistical significance was defined as a *p* value < 0.05. All statistical analyses were performed using Python version 3.11 (Python Software Foundation) and the stats models library (v0.13).

## RESULTS

At the final follow‐up, 310 patients were evaluated with a mean follow‐up period of 2.93 ± 0.62 years.

The cohort included 181 females (58.4%) and 139 males (41.6%), with an average age of 69 ± 8.3 years. A total of 167 right knees (53.9%) and 143 left knees (46.1%) were treated. The mean BMI was 28.6 ± 4.9 kg/m^2^. Among the patients, a CS liner was used in 107 cases (34.5%), while a PS liner was used in 203 cases (65.5%).

At the preoperative clinical evaluation, the mean KSS‐knee score was 64.5 ± 13.1, and the KSS‐function score was 67.9 ± 15.3. Regarding the ROM data, the mean recurvatum was 0.6 ± 2.2°, the mean flexion contracture was 2.2 ± 4° and the mean maximum flexion was 119.3 ± 11.8°.

In the preoperative radiographic assessment, the mean mHKA was 174.1 ± 5.5°, the LDFA was 89.3 ± 5.8°, the MPTA was 86.3 ± 3.1° and the TS was 7.4 ± 3.1°. The Mako data on tibial component positioning showed an average varus alignment of 3.3 ± 1.7° and an average slope of 0.8 ± 0.8°. For the femoral component, the mean valgus was 0.5 ± 1.8°, the average implant flexion (ΔDFF) was 6.9 ± 2.6° and the mean external rotation was 0.2 ± 1.85°. The average ΔTS was 6.6 ± 3.1°. The mean CF was 7.7 ± 2.5°, and the mean ΔCF was 0.3 ± 3.8°.

At the final follow‐up, 21 major complications were reported, corresponding to 6.8% and representing the reintervention rate. Specifically, 13 patients (4.2%) underwent manipulation under anaesthesia or arthroscopic arthrolysis within the first 6 months after the first surgery due to stiffness, 3 patients (1%) underwent surgery for revision of the surgical scar, 2 patients (0.6%) were reoperated on for acute infection using the DAIR technique (debridement, antibiotics and implant retention), 1 patient (0.3%) underwent reoperation for revision of the femoral component, 1 patient (0.3%) underwent revision of the patellar component and 1 patient (0.3%) underwent a complete revision due to previously unknown nickel allergy.

The final implant survival rate at the final follow‐up was 99%, with 1% of patients undergoing partial or total implant revision.

### CF of the implant

Group A comprised 124 patients (40.0%), and Group B included 186 patients (60.0%). In the comparison between these two groups, no statistically significant differences were found in terms of mean follow‐up (*p* = 0.56), age (*p* = 0.11), side (*p* = 0.07) and BMI (*p* = 0.09). The type of liner used showed a significant difference (*p* = 0.04), with the CS liner used in 34 patients (27.4%) in Group A and in 73 patients (39.3%) in Group B. Additionally, gender also showed a statistically significant difference (*p* < 0.001), with 48 women (38.71%) in Group A and 133 women (71.51%) in Group B.

No preoperative differences were observed in terms of clinical scores, specifically in KSS‐knee (*p* = 0.08), KSS‐function (*p* = 0.35), recurvatum (*p* = 0.92) and flexion contracture (*p* = 0.24). A statistically significant difference was found in preoperative maximum flexion (*p* = 0.005), with Group A showing lower values (116.9 ± 11.6°) compared to Group B (120.8 ± 11.7°).

Similarly, the preoperative radiographic analysis revealed no statistically significant differences in mHKA (*p* = 0.06), LDFA (*p* = 0.11), MPTA (*p* = 0.97) and native TS (*p* = 0.29).

Regarding Mako data, there were no differences in tibial varus (*p* = 0.23), TS (*p* = 0.68) and femoral external rotation (*p* = 0.59). A statistically significant difference was observed in femoral valgus (*p* = 0.002), with Group A having a mean of 0.13 ± 2.06°, lower than 0.91 ± 1.65° in Group B.

**Figure 3:**
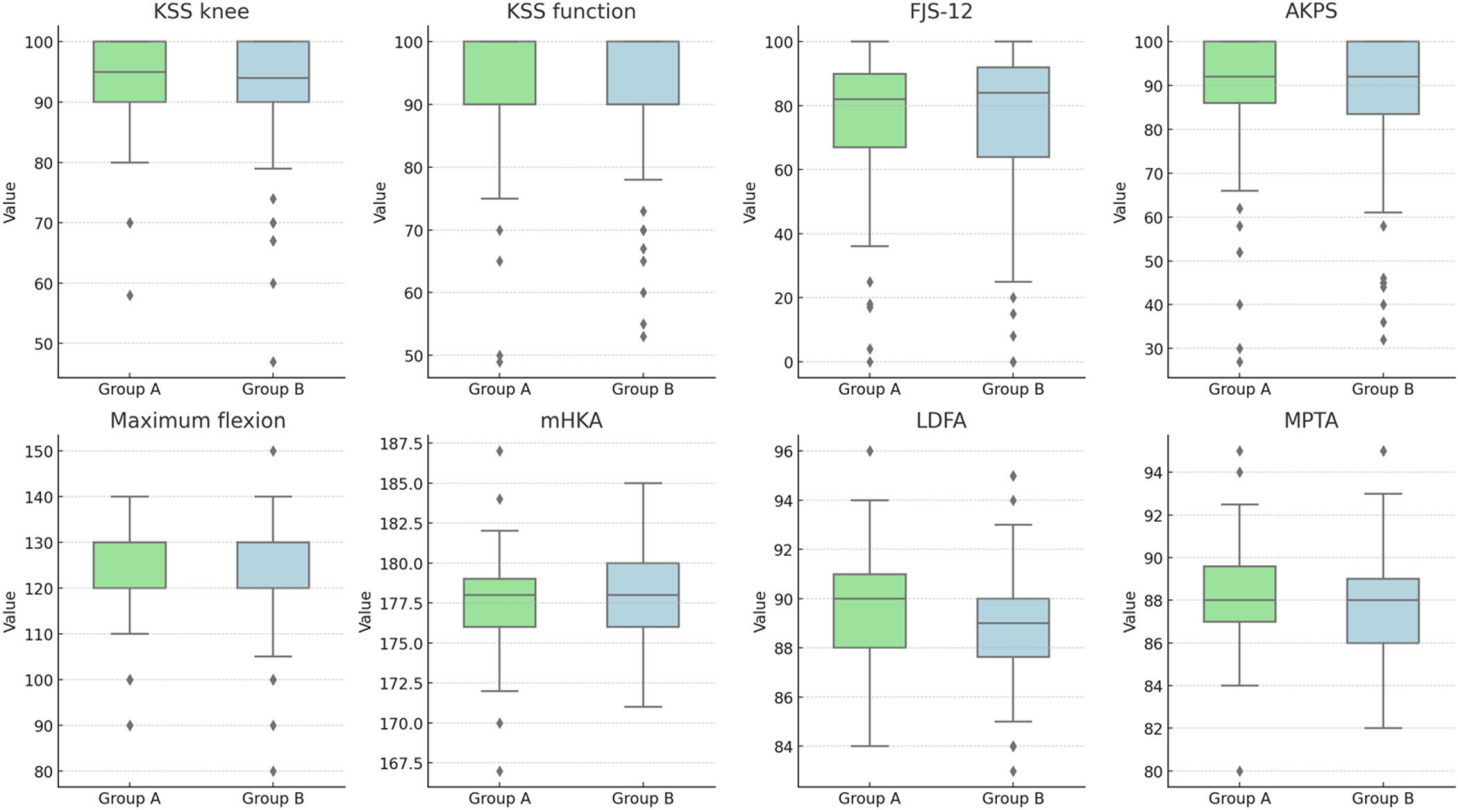
Graphical representation with box plots of the parameters analyzed in the comparison between Groups A and B, categorized based on CF, which is the sum of the tibial slope of the implant and the distal femoral flexion of the implant. Group A includes patients with CF ≤ 7.5°, while Group B includes patients with CF > 7.5°. AKPS, Kujala Anterior Knee Pain Scale; CF, combined flexion; FJS‐12, Forgotten Joint Score; KSS, Knee Society Score; LDFA, lateral distal femoral angle; mHKA, mechanical hip–knee–ankle angle; MPTA, medial proximal tibial angle.

At the final follow‐up, no differences were observed in clinical outcomes or radiographic measurements, except for post‐operative LDFA (*p* = 0.04). The details are provided in Table 1. Figure 3 shows the box plots of the analyzed parameters.

**Table 1:** Comparison of clinical outcomes and radiographic measurements at final follow‐up between Groups A and B, categorized based on CF, which is the sum of the tibial slope of the implant and the distal femoral flexion of the implant.

	Group A (*N* = 124)	Group B (*N* = 186)	*p* **value**
KSS knee	93.4 (SD 7.3)	92.4 (SD 8.7)	0.43
KSS function	92.1 (SD 9.4)	91.9 (SD 9.3)	0.88
Recurvatum (°)	1.3 (SD 2.9)	0.9 (SD 2.1)	0.47
Flexion contracture (°)	0.3 (SD 1.4)	0.2 (SD 1.0)	0.18
Maximum flexion (°)	123.5 (SD 10.2)	125.0 (SD 10.4)	0.15
FJS‐12	76.2 (SD 22.1)	76.8 (SD 21.9)	0.61
AKPS	89.4 (SD 13.6)	88.7 (SD 13.9)	0.81
mHKA (°)	177.6 (SD 2.9)	178.1 (SD 3.0)	0.20
LDFA (°)	89.7 (SD 2.4)	89.0 (SD 2.3)	0.04
MPTA (°)	88.2 (SD 2.1)	87.9 (SD 2.5)	0.20

*Note*: Group A includes patients with CF ≤ 7.5°, while Group B includes patients with CF > 7.5°.

Abbreviations: AKPS, Kujala Anterior Knee Pain Scale; CF, combined flexion; FJS‐12, Forgotten Joint Score; KSS, Knee Society Score; LDFA, lateral distal femoral angle; mHKA, mechanical hip–knee–ankle angle; MPTA, medial proximal tibial angle; SD, standard deviation.

There is no statistically significant difference in the overall complication rate (5.6% vs. 7.5%; *p* = 0.68), nor specifically in the causes of mechanical failure (4.0% vs. 5.3%; *p* = 0.89), the reoperation rate (4.0% vs. 7.0%; *p* = 0.40) or the implant revision rate (1.6% vs. 0.5%; *p* = 0.72).

### Variation in combined flexion (ΔCF)

**Figure 4:**
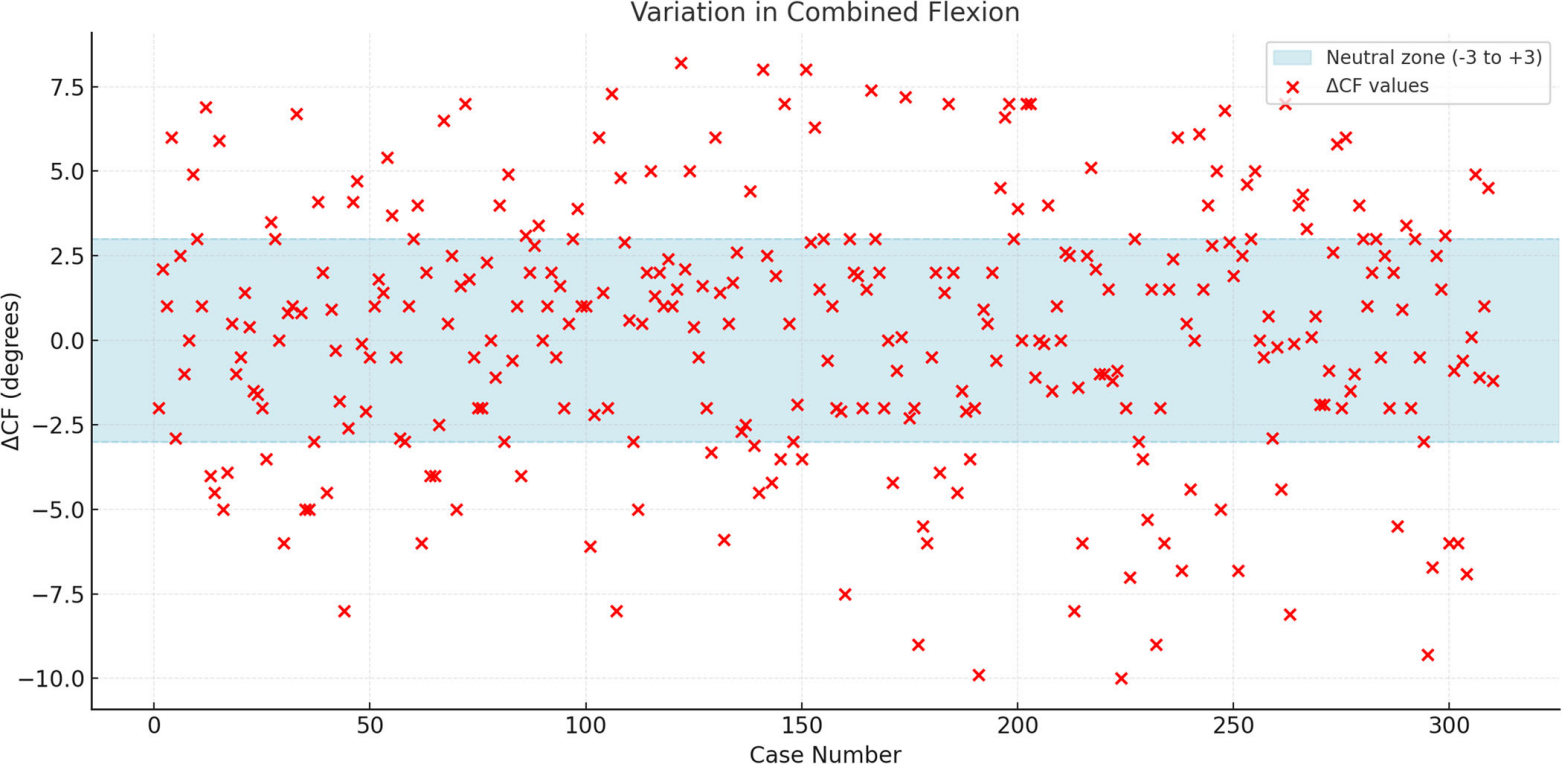
Distribution of the variation in combined flexion between native and implant flexion indicated as ΔCF.

Group C (ΔCF < −3) consisted of 54 patients (17.4%), Group D (ΔCF: −3 to 3) of 196 patients (63.2%) and Group E (ΔCF > 3) of 60 patients (19.4%). In Figure 4, the distribution of the ΔCF between native and implant flexion is represented.

No statistically significant differences were identified among the three groups concerning mean follow‐up (*p* = 0.17), gender (*p* = 0.42), age (*p* = 0.53), side (*p* = 0.57) or BMI (*p* = 0.14). The type of liner used showed no statistically significant difference (*p* = 0.10), with the CS liner used in 13 patients (24.1%) in Group C, 68 patients (34.7%) in Group D and 26 patients (43.3%) in Group E. Similarly, no preoperative differences were observed in KSS‐knee (*p* = 0.59), KSS‐function (*p* = 0.35), and range of motion (ROM; recurvatum: *p* = 0.44; flexion contracture: *p* = 0.84; maximum flexion: *p* = 0.35).

In the preoperative radiographic evaluation, there were no significant differences for mHKA (*p* = 0.09), LDFA (*p* = 0.15) or MPTA (*p* = 0.19). Regarding Mako data, no differences were found in tibial varus (*p* = 0.31), femoral external rotation (*p* = 0.27) or femoral valgus (*p* = 0.11).

At the post‐operative evaluation, in the presence of statistical significance in mHKA and MPTA, a post hoc analysis was performed. This analysis revealed a significant difference in mHKA between Groups C and D (*p* = 0.002) and between Groups C and E (*p* = 0.009), as well as a significant difference in MPTA between Groups C and D (*p* = 0.01).

**Figure 5:**
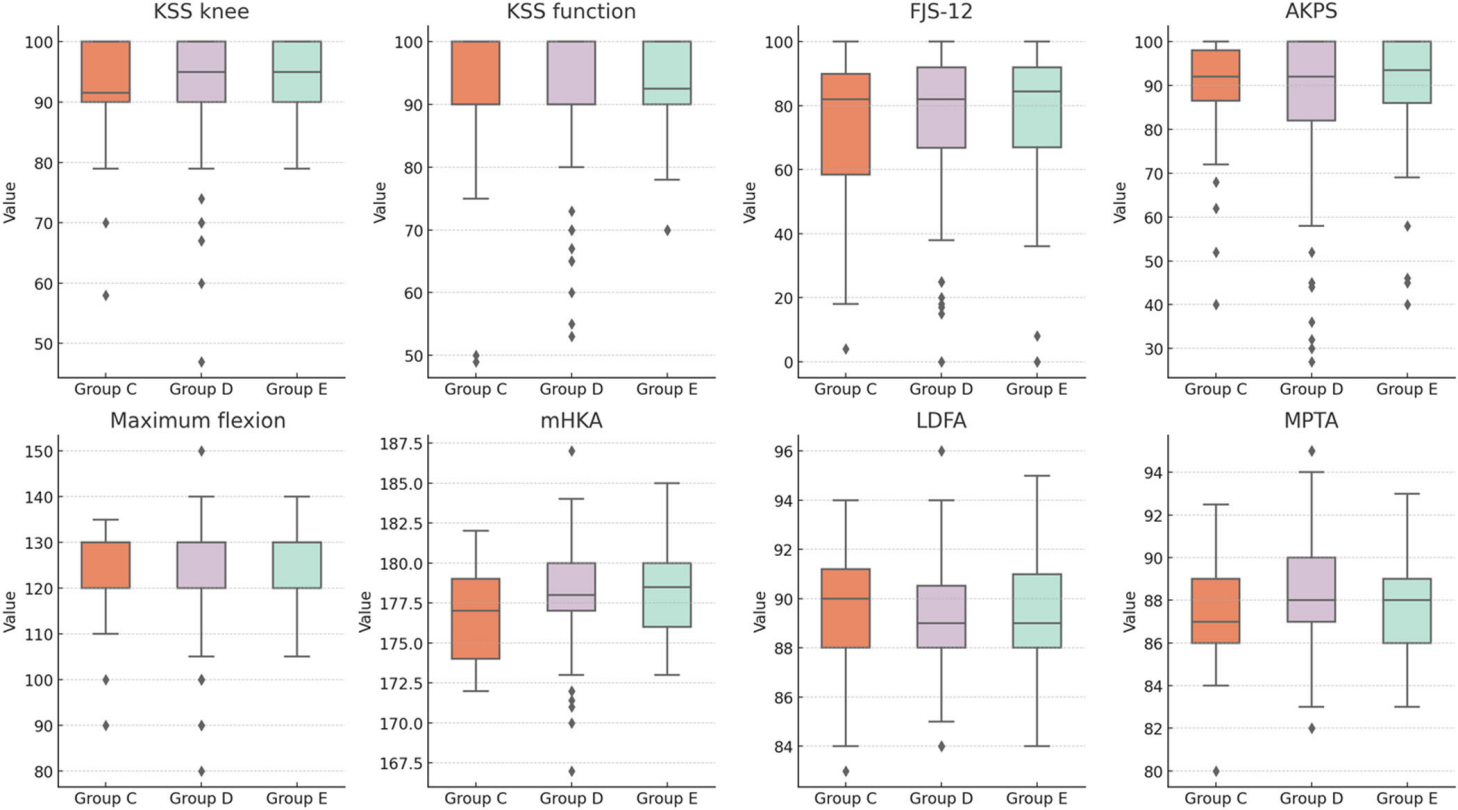
Graphical representation using box plots of the parameters analyzed in the comparison among Groups C, D and E, categorized based on ΔCF, which represents the variation between the native and final combined flexion (CF). Group C includes patients with ΔCF < −3, Group D includes patients with ΔCF between −3 and 3 and Group E includes patients with ΔCF > 3. AKPS, Kujala Anterior Knee Pain Scale; FJS‐12, Forgotten Joint Score; KSS, Knee Society Score; LDFA, lateral distal femoral angle; mHKA, mechanical hip–knee–ankle angle; MPTA, medial proximal tibial angle.

The details of the data analysis at the final follow‐up are provided in Table 2. Figure 5 shows the box plots of the analyzed parameters.

**Table 2:** Comparison of clinical outcomes and radiographic measurements at final follow‐up among Groups C, D and E, categorized based on ΔCF, which represents the variation between the native and final combined flexion (CF).

	Group C (*N* = 54)	Group D (*N* = 196)	Group E (*N* = 60)	*p* **value**
KSS knee	91.9 (SD 8.5)	92.8 (SD 8.4)	93.7 (SD 6.9)	0.57
KSS function	91.8 (SD 10.4)	91.8 (SD 9.3)	92.5 (SD 9.3)	0.91
Recurvatum (°)	1.5 (SD 3.2)	0.9 (SD 2.1)	1.3 (SD 2.6)	0.56
Flexion contracture (°)	0.3 (SD 1.6)	0.2 (SD 1.1)	0.2 (SD 0.9)	0.85
Maximum flexion (°)	123.9 (SD 9.1)	123.9 (SD 11)	126.4 (SD 8.8)	0.22
FJS‐12	74.1 (SD 21.9)	77 (SD 21.6)	77.3 (SD 23.3)	0.45
AKPS	89.3 (SD 12.4)	88.6 (SD 14.1)	89.9 (SD 13.7)	0.61
mHKA (°)	176.5 (SD 2.7)	178.2 (SD 3)	178.2 (SD 2.7)	0.005
LDFA (°)	89.7 (SD 2.7)	89.2 (SD 2.3)	89.4 (SD 2.2)	0.26
MPTA (°)	87.2 (SD 2.3)	88.3 (SD 2.3)	87.8 (SD 2.2)	0.03

*Note*: Group C includes patients with ΔCF < −3, Group D includes patients with ΔCF between −3 and 3 and Group E includes patients with ΔCF > 3.

Abbreviations: AKPS, Kujala Anterior Knee Pain Scale; FJS‐12, Forgotten Joint Score; KSS, Knee Society Score; LDFA, lateral distal femoral angle; mHKA, mechanical hip–knee–ankle angle; MPTA, medial proximal tibial angle; SD, standard deviation.

There is no statistically significant difference in the overall complication rate (9.3% vs. 6.1% vs. 6.7%; *p* = 0.73), nor specifically in the causes of mechanical failure (7.4% vs. 4.6% vs. 3.3%; *p* = 0.59), the reoperation rate (5.6% vs. 5.6% vs. 6.7%; *p* = 0.95), or the implant revision rate (3.7% vs. 0.5% vs. 0.0%; *p* = 0.07).

## DISCUSSION

The main findings of this study are that, following the principles of FA, a statistically significant difference was observed in gender distribution, with a higher percentage of females in the group with CF > 7.5°. In this group, significant differences also emerged in component positioning, with a greater valgus distal femoral cut. Despite these differences, at the final follow‐up, no statistically significant differences were found between the two groups in terms of clinical outcomes or complications.

Additionally, in the evaluation of ΔCF, statistically significant differences were found in post‐operative mHKA and MPTA values between the groups. However, no correlation was observed with clinical outcomes or complications.

Although no statistically significant differences were found among the analyzed groups, except for a decrease in maximum flexion in the group with reduced CF flexion, the literature suggests that CF plays a significant role in knee biomechanics.

The concept of sagittal alignment includes various aspects, among which is the importance of preserving anterior femoral offset (AFO) and posterior condylar offset (PCO), both of which are linked to femoral component flexion.

Wang et al. reported that personalized robotic‐assisted alignment achieved better restoration of AFO and PCO, with statistically significant differences compared to patients who received standard femoral sagittal alignment [35]. Additionally, at a 3‐month follow‐up, this translated into an improvement in the Hospital for Special Surgery Knee Score (HSS).

Preserving AFO is crucial as it can influence the biomechanical integrity of the knee joint, potentially leading to complications such as patellar malalignment and anterior knee pain [34]. Changes in anterior offset can result in over‐ or under‐stuffing of the patellofemoral joint, a well‐recognized factor contributing to revision surgery [4].

Similarly, maintaining PCO is essential for ensuring stability and range of motion after TKA, as variations greater than 2 mm have been associated with mid‐flexion instability [25]. Other studies have shown that increasing PCO can lead to tightness in the flexion gap or even a flexion‐extension mismatch [19, 21].

Chen et al. analyzed the impact of femoral component positioning at 0°, 3° and 6° of flexion in the sagittal plane [7]. They observed that a 3° flexion implantation could reduce excessive overhang, although 3.10 mm of overhang remained at the medial side of Zone 1. Conversely, 6° flexion could eliminate 3 mm of overhang across all zones but increased the risk of underhang. Thus, slight femoral flexion may be a useful technique to prevent excessive component overhang, particularly in the trochlea and anterior region of the distal condyle.

The desired tibial sagittal alignment in TKA is generally a posterior slope between 0° and 7°, which can be achieved either through bone resection or by incorporating the slope into the polyethylene insert. An increased TS is theorized to increase femoral rollback and improve knee flexion [14]. However, clinical studies did not find any statistically significant relationship between TS and knee kinematics, and no relationship between post‐operative knee movement and TS was observed [3, 5, 22].

With an increasing TS, there indeed is a decrease in tension within the PCL from a pure biomechanical point of view, as, in cruciate‐retaining (CR) TKA, lacking anterior cruciate ligament means that the femur will shift posteriorly, hence an increasing TS decreases distance from its origin toward PCL's point of insertion‐strains this soft tissue to the least level possible [20]. Since the CR designs are susceptible to abnormal kinematics such as paradoxical anterior sliding of the femur on the tibia, the role of the PCL in resisting anterior translation becomes very important. Excessive release of the PCL might result in paradoxical motion and could increase quadriceps muscle tension and patellofemoral pressure, both of which might be harmful for post‐operative recovery [8, 28].

Pan et al. described that, in patients undergoing TKA, with ΔTS > 3°, the medial tibiofemoral contact point was shifted anteriorly [27]. This is relevant because paradoxical motion following TKA may create mechanical problems with a reduction of quadriceps efficiency and altered biomechanics; this could have eventual consequences for clinical results [27].

Excessive TS can also shift the tibiofemoral contact point posteriorly, increasing the required quadriceps effort to extend the knee and creating a potential source of discomfort or instability with weight‐bearing activities [20]. When this contact point is moved too far posteriorly, it creates abnormal joint contact forces increasing compressive or tensile strain on the tibial weight‐bearing surface resulting in mid‐flexion discomfort [12]. Other studies have suggested that an increase in TS decreases the joint contact stress, lowers the quadriceps muscle force requirements during knee extension and minimizes posterior femoral impingement [18, 26].

In a study by Ersin et al., anterior‐posterior (AP) translation was evaluated, and no statistically significant correlation was found between KT‐1000 measurements and TS [10]. However, at 70° of knee flexion, a weak but significant correlation was observed, suggesting that AP translation decreases as TS increases. Based on these findings, the authors concluded that the optimal TS range for AP stability in TKA is between 4° and 6°, although no correlation was found between stability and patient satisfaction.

While changes in TS can influence knee kinematics and stability, the clinical impact remains uncertain. Although excessive TS may lead to abnormal AP translation and paradoxical motion, moderate TS adjustments could optimize stability and quadriceps efficiency in TKA.

This study has some limitations. The design is retrospective, and the follow‐up period is relatively short. The use of a single implant type and a single robotic system may limit the applicability of the results to other designs or platforms. The TS was measured on lateral short x‐rays. Furthermore, the inter‐ and intra‐observer reliability of radiographic measurements was not assessed. A more detailed analysis of variations of knee phenotypes should be conducted to obtain more personalized insights [16]. A ROC curve analysis was not performed to determine the cut‐off values. The follow‐up time, although relatively short, is sufficient for assessing short‐term outcomes but may limit considerations regarding complications. The radiographic evaluation did not include the assessment of radiographic signs of early loosening.

Despite these limitations, the study provides insights into sagittal alignment in robotic‐assisted TKA. Future research with longer follow‐up and advanced biomechanical analysis will help refine these findings and further clarify the long‐term impact of sagittal alignment variations on clinical outcomes and implant longevity.

## CONCLUSION

The role of the sagittal plane was also studied in the first part of this research, titled ‘Beyond the coronal plane in robotic total knee arthroplasty—Part 1: Variations in tibial slope and distal femoral flexion do not affect outcomes’ [2].

This second part of the study highlights that personalized sagittal alignment, following the principles of FA in robotic‐assisted TKA, allows for comparable results in terms of complication rates, overall clinical outcomes, and patient‐reported satisfaction, despite preoperative differences in maximum flexion.

Therefore, as observed in the first part of this study, it is evident that a personalized approach to sagittal alignment can safely be an integral component of functional knee positioning.

## AUTHOR CONTRIBUTIONS

Luca Andriollo and Sebastien Lustig had the idea for the article. Luca Andriollo was responsible for writing of the manuscript and qualified as corresponding author. Pietro Gregori was responsible for data acquisition and analysis and realisation of Figures and Tables. Christos Koutserimpas was responsible of statistical analysis. Cécile Batailler and Elvire Servien were responsible for conceptualisation and supervised data acquisition and analysis. Cécile Batailler, Elvire Servien and Sebastien Lustig were responsible for reviewing and critically revise the manuscript. All authors have given final approval of the version to be published.

## CONFLICT OF INTEREST STATEMENT

Cécile Batailler: Consultant for Smith and Nephew and Stryker. Elvire Servien: Consultant for Smith and Nephew. Sébastien Lustig: Consultant for Heraeus, Stryker, Depuy Synthes, Smith and Nephew. Institutional research support to Lepine and Amplitude. The remaining authors declare no conflicts of interest.

## ETHICS STATEMENT

This study adhered to the ethical principles outlined in the 1964 Declaration of Helsinki and complied with HIPAA regulations. Data collection and analysis were conducted in accordance with the MR004 Reference Methodology of the French Commission Nationale de l'Informatique et des Libertés (Ref. 2229975V0). All patients provided legitimate informed consent.

## Data Availability

The data that support the findings of this study are available from the corresponding author, [L.A.], upon reasonable request.
